# Acknowledgement of manuscript reviewers 2015

**DOI:** 10.1186/s12937-016-0137-1

**Published:** 2016-02-16

**Authors:** Nehme Gabriel

**Affiliations:** Central Florida Health Alliance, Lady Lake, USA

## Abstract

The editor of *Nutrition Journal* would like to thank all our reviewers who have contributed to the journal from Volume 14 (2015).

**Abdulla Badawy**

United Kingdom

**Adriana Franzese**

Italy

**Alain Koyama**

United States of America

**Albane Maggio**

Switzerland

**Alejandro Fernández Montero**

Spain

**Alexandra Jenkins**

Canada

**Alfredo Gea**

Spain

**Ali Alkan**

Turkey

**Ali Montazeri**

Iran

**Alison Coates**

Australia

**Amanda Patterson**

Australia

**Amanda Waddell**

United States of America

**Amedeo Lonardo**

Italy

**Ana Filipa Vicente**

United States of America

**Ana María Calderón de la Barca**

Mexico

**Anand Singh**

India

**Anand Tamatam**

India

**Andre Maltos**

Brazil

**Andrea Darling**

United Kingdom

**Andrew McKune**

South Africa

**Angelo Cagnacci**

Italy

**Anne-Louise Heath**

New Zealand

**Anthony Villani**

Australia

**Anwar-ul-Hassan Gilani**

Ethiopia

**Arcangelo Iannuzzi**

Italy

**Arianna Aceti**

Italy

**Arun Kumar Aggarwal**

India

**Asher Rosinger**

United States of America

**Ashok Tuteja**

United States of America

**Avula Laxmaiah**

India

**Babak Hooshmand**

Sweden

**Bagher Larijani**

Iran

**Barbara Dao**

Canada

**Barbara Rocha**

Portugal

**Barbara Taylor**

United States of America

**Bart Koot**

Netherlands

**Beat Müller**

Germany

**Bee Tan**

United Kingdom

**Beng San Yeoh**

United States of America

**Benjamin Nwosu**

United States of America

**Benoit Chassaing**

United States of America

**Bernd Weber**

Germany

**Biagio Raffaele Di Iorio**

Italy

**Bin Yu**

China

**Bing Wan**

China

**Bingjun Qian**

China

**Bonnie Beezhold**

United States of America

**Bryan Hackfort**

United States of America

**Caio Oliveira**

Brazil

**Carlos Pastor Vargas**

Spain

**Carol Johnston**

United States of America

**Catherine Chan**

Canada

**Chesney Richter**

United States of America

**Chris Frampton**

New Zealand

**Chris McGlory**

Canada

**Chris Rayner**

Australia

**Christine Sommer**

Norway

**Christine von Arnim**

Germany

**Christopher P. Morley**

United States of America

**Christopher Papandreou**

Greece

**Christopher Sudfeld**

United States of America

**Colin Rehm**

United States of America

**Connie Rogers**

United States of America

**Conrad Cole**

United States of America

**Corrine Hanson**

United States of America

**Creswell Eastman**

Australia

**Dagrun Engeset**

Norway

**Dallas Donohoe**

United States of America

**David Garcia**

United States of America

**David Jenkins**

Canada

**David Smith**

United Kingdom

**Dennis Nilsen**

Norway

**Diane Mitchelll**

United States of America

**Dick Chan**

Australia

**Dirk Aerenhouts**

Belgium

**Diwakar Bastihalli Tukaramrao**

United States of America

**Douglas Kalman**

United States of America

**Douglas MacKay**

United States of America

**Edel Rafael Rodea-Montero**

Mexico

**Eduardo De Stefani**

Uruguay

**Edward Levine**

United States of America

**Edward Randell**

Canada

**Elizabeth Pearce**

United States of America

**Emilie Combet**

United Kingdom

**Emma Patterson**

Sweden

**Emmanuel Grellety**

France

**Enzo Nisoli**

Italy

**Erick Boy**

Canada

**Esther Granot**

Israel

**Evan Cherniack**

United States of America

**Ewa Florek**

Poland

**Felipe Perez**

United States of America

**Frances Hillier-Brown**

United Kingdom

**Frank Greer**

United States of America

**Garden Tabacchi**

Italy

**Gerard Clarke**

Ireland

**Gerd Schmitz**

Germany

**Gerry Jager**

Netherlands

**Gilles Feron**

France

**Gilson Teles Boaventura**

Brazil

**Giorgina Piccoli**

Italy

**Giovanni de Pergola**

Italy

**Giovanni Tarantino**

Italy

**Giovanni Viegi**

Italy

**Girish Kirimanjeswara**

United States of America

**Giulia Marcelino Mainardi**

Brazil

**Giuseppe Grosso**

Italy

**Glen Lawrence**

United States of America

**Grace Farhat**

United Kingdom

**Guglielmo Trovato**

Italy

**H Zittermann**

Germany

**Hamed Kioumarsi**

Iran

**Hamid Reza Baradaran**

Iran

**Hanen Samouda**

Luxembourg

**Hanno Pijl**

Netherlands

**Hidetaka Hamasaki**

Japan

**Hiromichi Kumagai**

Japan

**Hiroshi Kaji**

Japan

**Hollie Raynor**

United States of America

**Howard Sesso**

United States of America

**Hui Sun**

United States of America

**I. Danquah**

Germany

**Ian Neeland**

United States of America

**Idoia Zarrazquin**

Spain

**Ilkka Heinonen**

Netherlands

**Imran Aziz**

United Kingdom

**Ingrid Kohlstadt**

United States of America

**Istvan Telessy**

Hungary

**Ita Litmanovitz**

Israel

**Ivana Strahinic**

Serbia

**J. Urbanek**

United States of America

**J. Wang**

United States of America

**Jacque Bay**

New Zealand

**Jairam Vanamala**

United States of America

**Jan Frank**

Germany

**Jean Abed**

United States of America

**Jessy Abraham**

India

**Jesus Vioque**

Spain

**Jin Han**

Republic of Korea

**JM He**

China

**JoAnn Long**

United States of America

**João Elias Nunes**

Brazil

**John Pettifor**

South Africa

**JON TORRES-UNDA**

Spain

**Jonathan Little**

Canada

**Joshua Lambert**

United States of America

**Julia Potter**

United Kingdom

**Julie Lumeng**

United States of America

**Julie Schmidt**

United Kingdom

**Julien Cases**

France

**Juscelino Tovar**

Sweden

**K Kinugawa**

Japan

**Kam-Lun Ellis Hon**

Hong Kong

**Kannan Sridharan**

Fiji

**Karin Schindler**

Austria

**Karthikeyan Subbiurayan**

India

**Katarina Bälter**

Sweden

**Kate Bowen**

United States of America

**Katherine Keene**

Canada

**Katherine Silva-Jaramillo**

Ecuador

**Kazuhiro Uenishi**

Japan

**Keiko Tanaka**

Japan

**Keiko Wada**

Japan

**Keiron Audain**

United Kingdom

**Keiron Audain**

Zambia

**Keng Fai Yip**

Malaysia

**Kevin Fritsche**

United States of America

**Kevin Maki**

United States of America

**Kim Sung-Hoon**

Republic of Korea

**Korry Hintze**

United States of America

**Kotatsu Maruyama**

Japan

**Kristina Tiainen**

Finland

**L Garneata**

United Kingdom

**Laila Hussein**

Egypt

**Laila Meija**

Latvia

**Laura Mullaney**

Ireland

**Lauri Polari**

Finland

**Leah Lipsky**

United States of America

**Leigh Ward**

Australia

**Leonardo Palombi**

Italy

**Leticia Elizondo-Montemayor**

Mexico

**Ligia Martini**

Brazil

**Lingjun Zhu**

China

**Lisa Jahns**

United States of America

**Liz Applegate**

United States of America

**Louise Purtell**

Australia

**Lucienne Lara**

Brazil

**Lun-Yi Zang**

United States of America

**Lynn Riddell**

Australia

**Lynne McFarland**

United States of America

**M Rizzo**

Italy

**M. Bonaccio**

Italy

**Makbule Gezmen-Karadag**

Turkey

**Manny Noakes**

Australia

**Maria Alicia Camina Martin**

Spain

**Maria Cristina Meri**

Italy

**Maria Jose Garcia-Meseguer**

Spain

**Maria Jose Romo-Palafox**

United States of America

**Maria Morales**

Spain

**Maria Pia Villa**

Italy

**Mariette Gerber**

France

**Marika Mikelsaar**

Estonia

**Mario Murcia**

Spain

**Marjanne Senekal**

South Africa

**Marjorie Freedman**

United States of America

**Marleen Baak**

Netherlands

**Marleen van Baak**

Netherlands

**Marta Van Loan**

United States of America

**Martin Ndegwa Mwangi**

Kenya

**Martin Root**

United States of America

**Martine Laville**

France

**Mary Ward**

United Kingdom

**Maryse Umugwaneza**

South Africa

**Masakazu Terauchi**

Japan

**Masataka Shiraki**

Japan

**Mat Krause**

United States of America

**Matthew Cannon**

United States of America

**Meagan Gillespie**

Australia

**Michael Donaldson**

United States of America

**Michael Holick**

United States of America

**Michael Rybak**

United States of America

**Michael Stamos**

United States of America

**Michelle Wien**

United States of America

**Ming Li**

Australia

**Mohamed Saleem**

India

**Monica H. Carlsen**

Norway

**Monica Millan**

Spain

**Monica Serra**

United States of America

**Mostafa Waly**

Oman

**MT Rotelli**

Italy

**Muraldihar Premkumar**

United States of America

**Muthalib Asha Murshida**

Sri Lanka

**Myriam Afeiche**

Switzerland

**Namson Lau**

Australia

**Naoki Ikegaya**

Japan

**Nasiruddin Khan**

Saudi Arabia

**Natalie Parletta**

Australia

**Nicolas Darcel**

France

**Nilesh Kashikar**

United States of America

**Nivedita Nagachar**

United States of America

**Norman Carvalho**

United States of America

**Olivia Phung**

United States of America

**Olusola Sotunde**

South Africa

**P Pedram**

Canada

**Pallavi Kawatra**

United States of America

**Pankaj Vashi**

United States of America

**Paola Gauffim Cano**

Argentina

**Paola Vitaglione**

Italy

**Pascale Fritsch**

United Kingdom

**Patricia Genaro**

Brazil

**Patricia Perez-Matute**

Spain

**Paul Jacques**

United States of America

**Paula Manuela Pereira**

United States of America

**Per Jeppesen**

Denmark

**Peter Celec**

Slovakia

**Peter Hannan**

United States of America

**Peter Konturek**

Germany

**Philippa Jackson**

United Kingdom

**Philippe Chauveau**

United States of America

**Pieter Jooste**

South Africa

**Pradeep Kumar**

United States of America

**Priscila Fassini**

Brazil

**Punam Ohri-Vachaspati**

United States of America

**Qian Bingjun**

China

**R Leon Guerrero**

Guam

**Rachel Brown**

New Zealand

**Rahim Aydin**

Turkey

**Rajesh Kumar**

India

**Ramón Serrano-Urrea**

Spain

**Rana Abu-Dahab**

Jordan

**Rangaiah Sashidharamurthy**

United States of America

**Ravi Dyavar**

United States of America

**Richard Bruno**

United States of America

**RM Hafizu**

United States of America

**Robin Tucker**

United States of America

**Roger Clemens**

United States of America

**Rok Orel**

Slovenia

**Rose Ann DiMaria-Ghalili**

United States of America

**Sabine Rohrmann**

Switzerland

**Sakae Miyagi**

Japan

**Samantha Kling**

United States of America

**Samuel Alaish**

United States of America

**Samuel Tchum**

Ghana

**Sandeep Prabhu**

United States of America

**Sandrine Péneau**

France

**Sanjay Mallya**

United States of America

**Sanne Kellebjerg Poulsen**

Denmark

**Santos Perez-Pozo**

Spain

**Sarah Garnett**

Australia

**Saverio Stranges**

United Kingdom

**Serenella Salinari**

Italy

**Sergio De Marchi**

Italy

**Sharon Erdrich**

New Zealand

**Shervin Assari**

United States of America

**Shetty Ravi Dyavar**

United States of America

**Shibig Yang**

China

**Shigeru Toki**

Japan

**Sibylle Kranz**

United Kingdom

**Sifan Chen**

Germany

**Simona Bo**

Italy

**Siwaporn Pinkaew**

Switzerland

**Skye McPhie**

Australia

**Søren Gregersen**

Denmark

**Søren Toubro**

Denmark

**Stéphane Duboux**

Switzerland

**SubbaRao Madhunapantula**

India

**Sumangala Gokavi**

United States of America

**Suresh Kumar Karri**

India

**Suresh Kumar**

India

**Susan Raatz**

United States of America

**Taisun Hyun**

South Korea

**Takuya Yoshida**

Japan

**Tamar Ringel-Kulka**

United States of America

**Tamatam Anand**

India

**Tania John**

Canada

**Tarig Merghani**

Saudi Arabia

**Teruo Kawada**

Japan

**Thiago Bruder-Nascimento**

Brazil

**Thomas Remer**

Germany

**Thomas Wolever**

Canada

**Tiange Wang**

United States of America

**Tina Jafari**

Iran

**Ujjawal Gandhi**

United States of America

**Ursula Schwab**

Finland

**Valeria Del Balzo**

Italy

**Venkateswarlu Chamcha**

United States of America

**Veronica Mocanu**

Romania

**Vicente Lahera**

Spain

**Vijay Ganji**

United States of America

**Vishal Singh**

India

**Vivek Narayan**

United States of America

**W Ma**

United States of America

**Wei Tang**

Japan

**Wen Liu**

United States of America

**Wenbo Tang**

United States of America

**William Grant**

United States of America

**William Manzanares**

Uruguay

**Wojciech Wojciech**

Poland

**Xu Tian**

China

**yasmin asif**

Pakistan

**Yehuda Shoenfeld**

Israel

**Yeyi Zhu**

United States of America

**Yiping Ma**

China

**Yong Zhu**

United States of America

**Yuan Zhou**

United States of America

**Yujie Wang**

United States of America

**Yulong Yin**

China

